# *Et tu, Neisseria*? Conflicts of Interest Between *Neisseria* Species

**DOI:** 10.3389/fcimb.2022.913292

**Published:** 2022-06-24

**Authors:** Rene Baerentsen, Christoph M. Tang, Rachel M. Exley

**Affiliations:** Sir William Dunn School of Pathology, University of Oxford, Oxford, United Kingdom

**Keywords:** *Neisseria*, pathogen, commensal, antagonism, growth inhibition

## Abstract

*Neisseria meningitidis* and *Neisseria gonorrhoeae* are two obligate human pathogens that have evolved to be uniquely adapted to their host. The meningococcus is frequently carried asymptomatically in the nasopharynx, while gonococcal infection of the urogenital tract usually elicits a marked local inflammatory response. Other members of the *Neisseria* genus are abundant in the upper airway where they could engage in co-operative or competitive interactions with both these pathogens. Here, we briefly outline the potential sites of contact between *Neisseria* spp. in the body, with emphasis on the upper airway, and describe the growing yet circumstantial evidence for antagonism from carriage studies and human volunteer challenge models with *Neisseria lactamica*. Recent laboratory studies have characterized antagonistic mechanisms that enable competition between *Neisseria* species. Several of these mechanisms, including Multiple Adhesin family (Mafs), Two Partner Secretion Systems, and Type VI secretion system, involve direct contact between bacteria; the genetic organisation of these systems, and the domain structure of their effector molecules have striking similarities. Additionally, DNA from one species of *Neisseria* can be toxic to another species, following uptake. More research is needed to define the full repertoire of antagonistic mechanisms in *Neisseria* spp., their distribution in strains, their range of activity, and contribution to survival *in vivo*. Understanding the targets of effectors could reveal how antagonistic relationships between close relatives shape subsequent interactions between pathogens and their hosts.

## Introduction

*Neisseria meningitidis* and *Neisseria gonorrhoeae* are closely related bacteria which are well-known human pathogens. *N. meningitidis* is a leading cause of community acquired sepsis and meningitis, particularly in children and young adults (Nadel and Ninis, 2018). Despite being a feared pathogen, *N. meningitidis* colonises the upper respiratory tract of healthy individuals without causing symptoms; only a small fraction of people who become colonised with the meningococcus subsequently go on to develop invasive disease. The bacterium can interact with epithelial cells *via* Type four pili, which allow initial attachment, mediate signaling and promote the formation of bacterial aggregates referred to as microcolonies (Pujol et al., 1997).

In contrast to the meningococcus, acquisition of *N. gonorrhoeae*, which typically colonises the urogenital tract, often results in local symptoms indicative of an inflammatory response (Hobbs et al., 2011). The development of a purulent discharge and/or dysuria usually prompts treatment with antibiotics, and eradication of infection after a few days (Unemo et al., 2019). In addition, asymptomatic infection with *N. gonorrhoeae* can also occur in both males and females (Chang et al., 2020).

Both species are exquisitely adapted to humans and are not found in any other host or in the external environment. Therefore, the evolution and virulence of both pathogens will be shaped by the environments they encounter in infected individuals. The microbiome is an aspect of the host environment which is receiving increasing attention because of the emerging evidence that it has a major impact on health and illness (Lynch and Pedersen, 2016). Within the gastrointestinal tract, there is clear evidence that the microbiome can exclude pathogens by competing for common host resources including nutrients and receptors. In addition, bacteria can gain an advantage over competitors occupying the same site by direct antagonism (Sassone-Corsi and Raffatellu, 2015). Far less is known about the interaction between bacteria at other body sites.

Aside from *N. meningitidis* and *N. gonorrhoeae*, several other members of the genus *Neisseria* are carried by humans (Liu et al., 2015) so have the potential to interact with the meningococcus and gonococcus. As discussed in this review, little is known about the co-existence and community behaviour of these different species, or whether competition has any role in shaping colonization and pathogenesis. However, it is now apparent that members of this genus possess tools with potential to antagonize their close neighbors. Here we review current knowledge about the sites colonised by both pathogenic and commensal species of *Neisseria*, highlight relevant epidemiological studies that point to potential antagonism between members of this genus in the upper airways, and detail evidence of antagonistic factors produced by *Neisseria* spp. We also discuss future avenues for research and discuss roles that competitive interactions between these related bacteria might play in shaping pathogen biology.

## *Neisseria* in the Human Host

The duration of *N. meningitidis* carriage has been examined in a few longitudinal studies, which indicate that any given strain can persist in a host for a period of over several months (Glitza et al., 2008). The rate of carriage is markedly affected by the host’s age, housing and other social behaviors. The proportion of carriers rises in teenagers from low levels in childhood (around 1-2%) to peak levels (around 20-40%) between the ages of 20-24 years (Cartwright et al., 1987; Caugant et al., 1994). Overcrowding and living in cramped conditions are associated with increased meningococcal carriage, as seen in students and military recruits (Glover, 1918; Neal et al., 2000). Other factors consistently associated with increased carriage include male sex, active/passive smoking, and recent kissing (Maclennan et al., 2006).

The non-pathogenic *Neisseria* spp. are also residents of the upper airway (Liu et al., 2015). Overall, the most prevalent genera found in the upper respiratory tract are *Streptococcus*, *Fusobacterium*, and *Prevotella* (Kumpitsch et al., 2019). However, microbiome studies using 16S rRNA amplification and sequencing have highlighted the presence of *Neisseria* spp. at multiple sites in the nasopharynx and oral cavity, as the fourth most abundant genus (Ramos-Sevillano et al., 2018) suggesting there is opportunity for *Neisseria* spp., to interact with each other in this niche. However, a significant limitation of this is that 16S sequencing lacks the discriminatory power to distinguish between *Neisseria* species. As a consequence, the overwhelming majority of studies that have examined human colonization with *Neisseria* spp. have relied on culture and characterization of bacteria. Although this allows definitive species identification, the initial recovery of *Neisseria* from samples usually relies on plating to ‘selective’ media (*i.e.*, containing agents such as colistin) to allow growth of *N. meningitidis* (the subject of most colonization studies) but suppress growth of other members of the flora. This reduces sensitivity and can introduce bias in results; for example, species including *Neisseria cinerea, Neisseria sicca*, *Neisseria perflava*, and *Neisseria mucosa*, are sensitive to agents such as colistin, so might not be recovered (Sáez Nieto et al., 1998; Clark et al., 2021). Specific methods have been developed to detect species such as *N. cinerea*, which was found in the nasopharynx of over 28% of healthy individuals attending a sexual health clinic in San Francisco (Knapp and Hook, 1988). In addition, *N. mucosa*, *N. sicca*, *N. subflava, Neisseria lactamica*, *Neisseria polysaccharea, Neisseria bergheri* and possibly novel *Neisseria* species have been isolated from the pharynx/oropharynx using culture-based approaches (Sheikhi et al., 2015; Diallo et al., 2016; Diallo et al., 2019; Calder et al., 2020).

A study of 40 healthy adults examined the co-colonization of different *Neisseria* species during nasopharyngeal carriage (Sáez Nieto et al., 1998). Virtually all participants carried *N. perflava-sicca* with 45% of people carrying at least one additional member of *Neisseria* spp. Of those carrying *Neisseria* spp., pulsed-field gel electrophoresis of isolates revealed that just over half were colonised by multiple strains of the same species. This is an important finding given that even members of the same species can have different antagonistic potential (*e.g.*, Type VI secretion systems, see below). This work provides evidence of the co-existence of multiple species within the same person, so there is scope for interaction between species and strains.

Evidence of co-colonization was confirmed in an important study that analyzed data from the Human Metagenomic Project (HMP) for the presence of *Neisseria* in the upper airway from 520 healthy individuals aged 18-40 years (Donati et al., 2016). The use of sequence data from the HMP circumvents limitations of culture-based methods, and also has the advantage that samples were taken from multiple sites in the oropharynx, providing some spatial resolution of the distribution of *Neisseria.* The authors assembled core genomes for each species from the metagenomic data to determine their presence in samples. Interestingly, there is clear evidence of tissue tropism for different *Neisseria* spp. in the oropharynx. For example, samples from the surface of the tongue were enriched for *Neisseria flavescens* and *N. subflava*, while gingival plaque harbored a combination of *N. mucosa, N. sicca*, and *Neisseria macacae*. These sites contained the most abundant and diverse populations of *Neisseria*, while longitudinal samples revealed stable colonization with the same strain on the dorsum of the tongue, similar to the longer-term carriage observed for *N. meningitidis*. Of note, *N. meningitidis* was not prevalent in this population and was predominantly found in the pharynx. Unfortunately, there were fewer samples from this site compared with the oral cavity so further work is needed to define the community of *Neisseria* spp. that co-exist with the meningococcus in the throat.

Work in the early 1970s showed that the upper respiratory tract is also an occasional home for *N. gonorrhoeae* (Bro-jorgensen and Jensen, 1973; Wiesner et al., 1973). This bacterium is the causative agent of gonorrhoea and provokes an acute inflammatory response in the urogenital tract, its usual residence in humans. However, the gonococcus can also be isolated from the oropharynx, with carriage often associated with infection at other body sites, such as the genitourinary tract (Peters et al., 2011). Usually, the gonococcus does not cause symptoms when found in the upper respiratory tract, but it still poses a threat due to the ability of obtaining resistance mutations from the commensal *Neisseria* community (Spratt et al., 1992; Wadsworth et al., 2018; Fiore et al., 2020).

In contrast to the upper airway, *Neisseria* spp. are not a significant component of the microbiome of the genitourinary tract, with most information available for the vaginal flora. Based on early studies, which relied on limited culture-based recovery and identification, some commensal *Neisseria* spp. such as *Neisseria catarrhalis*, *N. sicca*, *N. flava*, and *N. lactamica* have been occasionally recovered from the genital tract, where they generally cause no local symptoms (Wilkinson, 1952; Johnson, 1983). For example, at one sexual health clinic, *N. lactamica* was only isolated once from over 20,000 patients (Telfer Brunton et al., 1980). Instead, *Lactobacillus* predominates in the vaginal microbiota, and shares this niche with other genera including *Gardnerella*, *Atopobium*, *Prevotella*, and *Streptococcus* (Ravel et al., 2011). The low pH of the female vagina is generated by acid-producing strains of *Lactobacillus* and is likely detrimental for the survival of *N. gonorrhoeae* and other *Neisseria* spp. (Graver and Wade, 2011). Therefore, an important factor for genitourinary infection with the gonococcus might be disruption of the healthy microbiome, as seen in conditions such as bacterial vaginosis (Fredricks et al., 2005). Far less is known about the flora of the urethra in males, even though it might influence whether exposure to the gonococcus leads to acquisition and the development of disease. Of note, *N. meningitidis* sometimes causes a clinical syndrome which is indistinguishable from gonorrhoea, with purulent urethritis and/or cervicitis; outbreaks of meningococcal urethritis have been documented in high-risk populations (for review see (Humbert and Christodoulides, 2019). Interestingly, the meningococcal strains that cause symptomatic urogenital disease share certain features, such as loss of capsule expression and changes in respiratory pathways that might enable them to adapt to this niche (Tzeng et al., 2017).

In summary, current scientific knowledge suggests that the upper airway is the predominant niche where *Neisseria* species may encounter one another. However, although carriage surveys have indicated the prevalence of different *Neisseria* species among populations and age groups, further unbiased sequence-based studies of co-colonization of the nasopharynx and urogenital tract by *Neisseria* species and strains are required for a clearer picture of the relevance and frequency of multi-species and multi-strain colonization which can guide studies on bacterial antagonism.

## Insights Into Relationships Between *Neisseria* Species From Epidemiological Studies: Challenging Concepts

Current insights into relationships between *Neisseria* species in vivo have been gained from studies showing the age-related, inverse association of carriage of *N. lactamica* and *N. meningitidis*, and observations from human challenge experiments (Gold et al., 1978; Evans et al., 2011; Deasy et al., 2015). *N. lactamica* is perhaps the best characterized species among the non-pathogenic *Neisseria* species and has been investigated largely for its potential to elicit protective immune responses against meningococci, both through colonization (Gold et al., 1978) and by its use as a potential vaccine, either as *N. lactamica* derived outer membrane vesicles (Gorringe et al., 2009) or more recently as a live, recombinant strain for delivery of meningococcal antigens (Laver et al., 2021). *N. lactamica* is frequently carried asymptomatically in the nasopharynx of infants and children (Gold et al., 1978; Kristiansen et al., 2012; Diallo et al., 2016). Gold et al., observed that in the first four years of life, 59% of children have carried *N. lactamica* at least once (Gold et al., 1978). On the other hand, carriage of meningococcus is less frequent in these younger age groups (Gold et al., 1978; Holten et al., 1978; Gelosa, 1981; Saez-Nieto et al., 1985; Cartwright et al., 1987; Diallo et al., 2016). Interestingly, in older age groups the inverse pattern is observed *i.e.*, in young adults, meningococcal carriage is prevalent, but *N. lactamica* is less frequent (Gold et al., 1978; Kristiansen et al., 2012; Diallo et al., 2016). This epidemiological relationship has been proposed to be due to *N. lactamica* inducing cross protective humoral immunity against meningococcus (Gold et al., 1978; Evans et al., 2011). The ability of the meningococcus but not *N. lactamica* to utilize propionate produced by anaerobes that are enriched in the adult upper airway microbiome has also been proposed as a possible explanation (Catenazzi et al., 2014). Experimental human infection experiments have provided further insight into carriage of *N. lactamica* and the relationship with carriage of *N. meningitidis*. In a study by Evans et al, in which adult volunteers aged 18-45 years were experimentally inoculated with *N. lactamica*, over 60% became colonised. Some individuals sustained colonization for 24 weeks, demonstrating that this organism can establish colonization of adults (Evans et al., 2011). Notably however, none of 26 individuals who were colonised after inoculation with *N. lactamica* acquired *N. meningitidis* throughout the duration of the study, while three out of 20 control subjects did. Although an increase in cross reactive anti-meningococcal opsonophagocytic responses (which significantly increased in colonised individuals) could account for this, immunization with *N. lactamica* OMVs did not prevent *N. meningitidis* carriage, leading the authors to propose that the presence of *N. lactamica* in the nasopharynx, rather than any subsequent immune responses, may inhibit acquisition of the meningococcus. In a subsequent study, 149 18–25-year-old university students were inoculated with *N. lactamica* (Deasy et al., 2015); by two weeks after inoculation 33% were colonised by the commensal species and carriage rates remained around 20-30% over 26 weeks. Importantly, carriage of *N. meningitidis* increased over time in the control group, but there was a significant reduction in meningococcal carriage in the participants who were colonised with *N. lactamica*. Furthermore, in individuals who were carriers of meningococcus at the time of inoculation, there was a greater reduction of *N. meningitidis* carriage after two weeks in those who became colonised by *N. lactamica* compared to those who were inoculated but not colonised. This indicates that colonization with *N. lactamica* could both prevent acquisition of the pathogen and displace existing *N. meningitidis* (Deasy et al., 2015). Similarly, colonization by *N. lactamica* following challenge was lower in individuals carrying *N. meningitidis* at the time of challenge compared with those who were not colonised by meningococcus, suggesting that carriage of meningococcus could inhibit colonization by *N. lactamica* (Deasy et al., 2015). These findings suggest that presence of either species can impact successful establishment of colonization of the human nasopharynx by a closely related organism, a concept that is consistent with the low frequency of co-colonization by both organisms reported by others (Simmons et al., 2000; Cleary et al., 2016).

## Antagonism Between *Neisseria* Species During *in Vitro* Growth

Currently only a few studies have explored competitive interactions between *Neisseria* species *in vitro*. The commensal *N. cinerea* has been shown to impact association of the meningococcus with human epithelial cells; both pre-infection of cells with *N. cinerea* followed by *N. meningitidis*, or simultaneous infection with both species in an equal ratio leads to a relative reduction in the association of the pathogen with cells, compared to single species infections (Custodio et al., 2020). It is not clear what underlies this observation. In other studies, intra- or inter-species competition in *Neisseria* has been examined in the context of specific factors predicted to function as inhibitory molecules, such as polymorphic toxins, bacteriocins and more recently methylated DNA as discussed below.

### Polymorphic Toxins and Their Delivery

Inhibitory interactions between bacteria can be mediated by secreted or translocated proteinaceous polymorphic toxins. These mediators of bacterial antagonism follow some general design principles; they consist of components necessary for secretion, a toxin, and an immunity protein. The toxin is generally a multidomain protein with the N-terminal domain implicated in secretion and the C-terminal domain mediating the toxic activity (Ruhe et al., 2020). Usually, the gene immediately downstream of the toxin-encoding gene codes for the immunity protein. These immunity proteins prevent self-intoxication, but also confer immunity against related toxins secreted by kin (Zhang et al., 2012).

**Figure 1:**
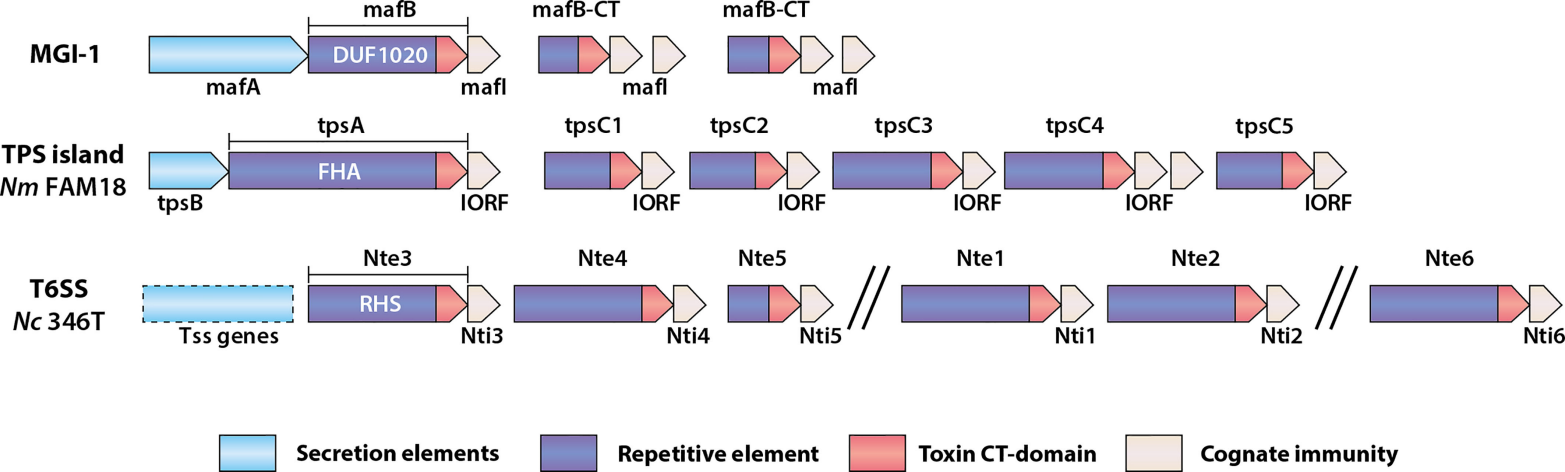
Schematic of genomic organization of polymorphic toxin system loci found in *Neisseria* species. Examples of a Maf genomic island, a TPS island and T6SS associated effectors are shown. MGI-1 is the consensus organization of this island (Arenas et al., 2015; Jamet et al., 2015), whereas the TPS island and T6SS locus indicated are from *N. meningitidis* (Nm) FAM18 (Arenas et al., 2013) and *N. cinerea* CCUG346T (Custodio et al., 2021), respectively. The secretion elements of the Maf and Tps toxins are different between systems but are generally located in the 5’-end of the genomic islands. *mafB* includes a region encoding a domain of unknown function named DUF1020, *tpsA* includes a region encoding a filamentous hemagglutinin adhesin (FHA) domain, whereas T6SS effector genes like those described in *N. cinerea* (Nc) include regions encoding Rearrangement Hot Spot (RHS) domains. The repertoire of toxin genes is diverse, but each is followed by a gene encoding a cognate immunity protein.

The MafABI system, which constitutes a novel group of polymorphic toxins in *Neisseria* spp., contains the outer membrane-associated protein MafA, the two-domain toxin MafB, and the immunity protein MafI (Arenas et al., 2015; Jamet et al., 2015). *maf* genes are prevalent in pathogenic *Neisseria* and are estimated to comprise about 2% of the genome (Jamet et al., 2015). In *N. meningitidis* and *N. gonorrhoeae, maf* genes are organized into five *maf* genomic islands (MGI-1 to MGI-5) which are classified based on conserved locations in the genomes. MGI-1, -2 and -3 are found in meningococci and gonococci, while MGI-4 and -5 are only present in gonococcal genomes. A scheme of a *maf* island (MGI-1) is shown in **Figure 1**. All MGIs contain a *mafA* gene but differ in the number of full-length *mafB* and *mafI* genes. MGIs also harbor a variable number of truncated *mafB* genes with cognate *mafI* genes. This feature, of having multiple copies of genes encoding C-terminal toxin domains with a cognate immunity protein, is common to loci for several polymorphic toxins (**Figure 1**). *maf* genes also exist in commensal *Neisseria*. MGI-1 is found in *N. cinerea* (ATCC 14685) and *N. polysaccharea* (ATCC 43768), *N. lactamica* 020-06 has MGI-1, MGI-3 and frameshifted MGI-2 and MGI-5, and *N. flavescens* (NRL30031/H210) have a fragmented MGI-5. Based on the presence of both intact *mafA* and *mafB* in some of the MGIs they could potentially still be functional. In contrast, *N. elongata* (ATCC29315), *N. mucosa* (ATCC 25996 and C102) and *N. subflava* (NJ9703) do not appear to have *mafA* or *mafB* homologues (Jamet et al., 2015), although this does not exclude the presence of *maf* genes in other strains.

MafB toxins themselves comprise a conserved N-terminus which includes a domain of unknown function (DUF1020 domain) that is unique to *Neisseria* MafB toxins (**Figure 1**). They can be divided into three classes, denoted as MafB1-3, based on variation in specific motifs located at the end of the conserved N-terminal domain. The C-terminal toxin domains are highly variable, meaning that different MafB toxins likely have different modes of toxicity. MafB from MGI-1 in *N. meningitidis* 8013 (MafB_MGI-1NEM8013_) was shown to be an RNase with EndoU ribonuclease activity; this toxin can degrade RNA *in vitro*, an activity that is blocked by the immunity protein (Jamet et al., 2015). Bioinformatic analysis has indicated that MafB2_MGI-2Nm053442_ also possesses an EndoU nuclease fold domain (Zhang et al., 2011). However, little is known about the specific activities of other MafB toxins.

The secretion pathway of MafB has been investigated. Due to the presence of a conserved N-terminal signal sequence, the toxin is proposed to cross the inner membrane *via* the Sec system (Jamet et al., 2015). Original observations that MafB could be detected in culture supernatants in the absence of a MafA led to speculation that the toxins may be secreted by association with outer membrane vesicles (Jamet et al., 2015). A later study revealed that MafA mediates MafB translocation across the outer membrane, and after secretion MafB can undergo further maturation *via* auto-proteolysis of a HINT domain (Arenas et al., 2020). In contrast, the path of entry of secreted toxins into target cells is poorly understood and merits further study. There is also evidence showing that there is some degree of MafA selectivity during MafB secretion. Whereas MafB1 is only secreted by MafA from the same MGI, MafB3 can be secreted by MafA from other MGIs (Arenas et al., 2020). This selectivity could be important in interpreting the potential secretion of MafB toxins in *Neisseria*, since certain isolates have multiple MGIs, some of which lack MafA.

The MafABI system has been shown to contribute to bacterial competition, but not all MafB toxins mediate these effects; so far only MafB toxins from MGI-2 and -3 have been associated with growth inhibitory activity (Arenas et al., 2015; Jamet et al., 2015). Overexpression of the MafB toxin (MafB1_MGI-2NEM8013_) and its cognate immunity protein in strain NEM8013 enabled this strain to inhibit growth of an unencapsulated *N. meningitidis* NEM8013 devoid of the cognate *mafI* gene (Jamet et al., 2015). Interestingly, expression of this same MafB toxin from a plasmid in *E. coli* BL21(DE3) did not impact *E. coli* growth (Jamet et al., 2015); whether this is due to this MafB toxin having a *Neisseria* specific target, or the mode of delivery of MafB is unclear. It is also of note that growth inhibition was seen when the *Neisseria* target strain was unencapsulated, given that many carried meningococci lack the genes required for capsule synthesis and transport (Claus et al., 2002), and the down regulation of capsule is a key step in meningococcal pathogenesis (Tzeng et al., 2016). The presence of a capsule offers protection from polymorphic toxin-mediated growth inhibition in other important human pathogens such as *Acinetobacter baumannii* (Krasauskas et al., 2020).

Little is known about the roles of other MafB toxins. Therefore, elucidation of toxin activity, and further competition studies with other meningococcal lineages or *Neisseria* species is needed to provide insight into Maf system functions. Interestingly, MGI-4 and -5 are only present in gonococcal genomes (Jamet et al., 2015) and the bacterial transcriptional regulator NadR represses expression of *mafA* in the presence of 4-HPA, a molecule that is present in human saliva (Liguori et al., 2016), suggesting that MafB toxins may be species specific or adapted to particular niches. Currently all studies of the MafABI system have been conducted in *N. meningitidis*, meaning that almost nothing is known of this system’s involvement in antagonism in *N. gonorrhoeae* or commensal species.

In addition to MafB toxins, meningococci can express other polymorphic toxins, which belong to the family of two-partner secretion (TPS) systems. These are widespread in Gram-negative bacteria and encode distinct functions (Guérin et al., 2017), including toxins that can mediate inter-bacterial antagonism, also known as contact dependent inhibition (CDI) systems (Aoki et al., 2005). In general, CDI toxins are large, filamentous proteins that interact with, and are secreted by, a partner transporter *via* sequences in their N-terminus. The C-terminal (CT) domain is required for toxicity and is delivered to the target cell after cleavage from the secreted toxin (Ruhe et al., 2018). In different bacteria these domains are highly variable and have been shown to have different toxic capabilities, such as DNase or tRNAse activities. These systems often form loci that contain the transporter, toxin and a cognate immunity protein (Ruhe et al., 2020).

In *N. meningitidis*, TPS systems comprise the secreted toxin, generically known as TpsA, and the partner outer membrane transporter, TpsB (Van Ulsen et al., 2008; Arenas et al., 2013). Based on genome analysis meningococci can encode up to five TpsA toxins and two TpsB transporters (Van Ulsen et al., 2008). Similar to Mafs, the genes encoding the transporter and the toxins are located in islands. Also similar to *maf* islands, downstream of *tpsA* genes are multiple shorter *tpsA*-like genes known as *tpsC* cassettes as they encode for toxic domains without the regions necessary for secretion. Adjacent to them are genes referred to as IORFS (Interspacing Open Reading Frames) which confer immunity (Arenas et al., 2013) and **Figure 1**).

TpsA1 from *N. meningitidis* (also called HrpA) has been shown to have roles in adherence (Schmitt et al., 2007), intracellular replication (Talà et al., 2008), biofilm formation (Neil and Apicella, 2009), and can also act as a CDI toxin (Arenas et al., 2013). Experimental analysis has demonstrated that a wild-type *N. meningitidis* strain (FAM18) which expresses only a single TpsA toxin (TpsA1, **Figure 1**) can inhibit growth of an un-encapsulated isogenic mutant lacking the *tps* island when grown together on solid media (Arenas et al., 2013). Complementation of the mutant with IORF1 abolished the growth inhibition by the wild type, providing evidence that IORFS confer immunity. The contribution of the *tpsC* cassettes in the *tps* islands to growth inhibition is not clear. Given their shared sequence homology it was proposed that they act as donors for recombination with *tpsA*, allowing shuffling of the toxin domain and thereby altering the activity of the system (Arenas et al., 2013). However, although there is some evidence of recombination between *tpsC* and *tpsA* loci from genome analysis and *in vitro* experiments, the frequency of such events is low and may even be unfavourable, possibly by affecting the repertoire of immunity genes and rendering the bacterium susceptible to growth inhibition by kin (Arenas et al., 2013). Thus, the current hypothesis for the presence of multiple *tpsC*-IORFs in *tps* islands is to provide immunity against different TpsA toxins, rather than serve as sequences for generating new toxins (Arenas et al., 2013).

Another way to deliver polymorphic toxins is *via* a Type VI Secretion System (T6SS). In this system toxins are delivered after they are loaded onto a multiprotein “needle” that is assembled in the cytosol and fired into target cells (Basler et al., 2013). To eject this poison-tipped needle, a machinery is built that spans both membranes of the attacking cell (Pukatzki et al., 2007; Leiman et al., 2009). This injection machinery is evolutionary linked to bacteriophages that deliver their genomic payload into prey cells (Cianfanelli et al., 2016b). T6SS toxins (usually referred to as effectors) are multidomain proteins and are related to Rearrangement Hot Spot (RHS) proteins (Hill et al., 1994). The N-terminal regions of many T6SS effectors also comprise domains with homology to one of the three main parts of the needle, the tip (PAAR), head (VgrG), or shaft (Hcp) (Zhang et al., 2012; Whitney et al., 2014; Unterweger et al., 2015; Flaugnatti et al., 2016; Ma et al., 2017). However, some toxins do not obviously contain PAAR, VgrG or Hcp homologous regions, indicating that there might be other ways they associate with the needle. Like the Maf and TPS systems, the C-terminal domain of T6SS effectors carries the toxic component (**Figure 1**). Examples of toxic activities include nuclease (Pissaridou et al., 2018), peptidoglycan glycoside hydrolase (Whitney et al., 2013), ion-selective pore formation (Mariano et al., 2019), phospholipase (Flaugnatti et al., 2016) and NADase (Tang et al., 2018). The RHS domain between the N- and C-terminus contains several repeating motifs, which when folded form a β-sheet cage around the toxic domain (Günther et al., 2022). At the border between these three domains are dual autoproteolytic DPxGL motifs (Jurėnas et al., 2021). It is not clear when this cleavage occurs, but since the RHS domain forms a protective shell around the C-terminal toxin, and since the effector needs to be attached to the needle, which happens *via* the N-terminus, proteolysis is thought to occur after secretion (Jurėnas et al., 2021). Secretion of the toxin-loaded needle happens first by assembly of the basal plate spanning the inner membrane. This basal plate contains VgrG and PAAR and therefore also in some systems toxins which share homologous regions (Cianfanelli et al., 2016a). The construction of the needle is then formed by the progressive addition of Hcp sheathed by TssBC complexes (Cherrak et al., 2019). To fire the needle, ClpV, an ATPase, induces a conformational change in TssBC, pushing the needle through the outer membrane and into a nearby target cell, delivering effectors to the cytoplasm or periplasm (Cherrak et al., 2019). So, similar to the Maf and TPS systems, the mechanisms of secretion of toxins are partly understood, but questions remain about how they are delivered into target cells.

Among *Neisseria*, so far T6SS genes have only been identified in some genomes of commensal species but not all members of those species have equal potential for antagonistic effects (Marri et al., 2010; Calder et al., 2020; Custodio et al., 2021). To date only a single *Neisseria* T6SS has been experimentally characterized, found in an isolate of *N. cinerea*, CCUG346T (Custodio et al., 2021). In this strain, the genes for the secretion apparatus, putative effectors and cognate immunity proteins are all located on a large plasmid. The putative effectors have predicted DNAse, RNAase or phospholipase activities and, except for one, are located along with their cognate immunity gene in proximity to the T6SS structural components. Although toxicity of the effectors has been shown by expression in *E. coli* (Custodio et al., 2021) their targets, and individual impact upon growth of competing *Neisseria* has not been determined. Nevertheless, the presence of the T6SS confers an advantage, as demonstrated by the significant T6SS-dependent reduction in recovery of prey strains of either *N. cinerea* that lack the T6SS, and also *N. meningitidis* or *N. gonorrhoeae* in co-culture experiments (Custodio et al., 2021). These initial findings point to a role of T6SSs in intra- and inter- species antagonism. Elucidating how widespread these systems are and understanding the diversity of effectors and immunity elements within and between the species will be of interest for understanding the importance and potential roles of T6SSs in competition between *Neisseria* species.

### Antimicrobials Produced by *Neisseria* Species

Bacteriocins are antimicrobial peptides and proteins produced by bacteria. They are generally defined as ribosomally synthesised peptides, to differentiate them from non-ribosomally produced antimicrobial products. Bacteriocins comprise a diverse group of molecules including small peptides (less than 10 kDa) which can be broadly classified according to whether they are modified or not (reviewed in (Soltani et al., 2020)), as well as larger proteins such as colicins (Cascales et al., 2007; Kleanthous, 2010) or complexes such as phage tail-like bacteriocins (Scholl, 2017). Bacteriocins can target bacterial cell membranes resulting in lysis or can exploit specific receptors and outer membrane proteins for translocation into the periplasm or cytoplasm, where they can affect fundamental processes in target cells (Rebuffat, 2022). Bacteriocin production is widespread among bacteria and, due to their potent but relatively specific activity, these molecules have been proposed as alternatives to antibiotics (Cotter et al., 2013; Telhig et al., 2020). Producer strains have self-immunity *via* several possible mechanisms, for example by expression of cognate immunity proteins, similar to the polymorphic toxin-immunity pairs, or export pumps (for review see (Rebuffat, 2022)). While some bacteriocins display a broad target range, many exert their antimicrobial activity on organisms belonging to the same species or genus as the producer strain. Bacteriocins are diffusible molecules, hence their expression can provide the producing strain with a competitive advantage within bacterial communities, and bacteriocin production has been shown to impact niche competition in the gastrointestinal tract in animal models (reviewed in (García-Bayona and Comstock, 2018; Heilbronner et al., 2021). Consistent with a role in shaping complex bacterial communities, many of the species in the human microbiome have the capacity to produce bacteriocins (Drissi et al., 2015; Walsh et al., 2015; Zheng et al., 2015).

The potential production of bacteriocins by pathogenic *Neisseria* species has been explored in several studies. Early work on *N. gonorrhoeae* described the production of “gonocin”, following the observation that some strains inhibited growth of other gonococci, and that the growth inhibition phenotype had characteristics of bacteriocins defined at the time (Flynn and Mcentegart, 1972; Lawton et al., 1976). However, similar investigations identified gonococcal growth inhibitory substances that were proposed to be metabolites or fatty acids and lyso-phosphatidyl ethanolamine (Walstad et al., 1974); these had broad anti-gonococcal activity, including against producer strains, not consistent with an effect mediated by a bacteriocin. A similar but undefined inhibitory activity was also reported by Knapp *et al*., who suggested that a toxic metabolite rather than a bacteriocin was responsible and may have led to the conflicting reports of ‘gonocin’ production (Knapp et al., 1975). A meningococcal bacteriocin, ‘meningocin’ was first proposed in a study by Kingsbury which described an inducible (by mitomycin C or UV), heat stable, protease sensitive inhibitory activity against meningococci and some non-pathogenic *Neisseria* (*i.e.*, *N. flavescens*, *N. subflava* and *N. perflava*) (Kingsbury, 1966). Meningococcal growth inhibition against *N. gonorrhoeae* has also been described (Allunans and Bøvre, 1996); interestingly anti-gonococcal activity was also reported in a strain of meningococcus isolated from the urethra (Volk and Kraus, 1973). The first partial purification of a putative meningococcal bacteriocin led to the identification of the active component as a 47-48 kDa polypeptide (Allunans et al., 1998), suggestive of a colicin-type bacteriocin. However, the product was not sufficiently active or not available in sufficient amounts to allow further characterisation. Finally, two genes (NMB0097 and NMB0098) have been implicated in the secretion of a potential meningocin; homologues were not detected in any sensitive strains and deletion of either gene in a producer meningococcal strain abolished growth inhibition (Allunans et al., 2008), however the genes encoding the proposed meningococcal bacteriocin(s) have not been identified.

Despite the lack of any genetically and biochemically characterized *Neisseria* bacteriocin, these studies suggest that both *N. meningitidis* and *N. gonorrhoeae* have the capacity to produce antimicrobial substances that impair growth of related strains and other *Neisseria* spp. The presence of bacteriocin genes in genomes of *Neisseria* species in the oral cavity has been reported (Zheng et al., 2015) and with available whole genome sequences and online tools for identification of bacteriocin gene clusters (Walsh et al., 2015), there is scope to define the capacity of different *Neisseria* isolates to synthesise and secrete antimicrobial peptides, providing a better understanding of their antagonistic potential. Furthermore, the observation that both gonococci and meningococci have selective growth inhibitory properties against related strains or species, whether mediated by bacteriocins or other molecules, provides the foundation for future investigations. Indeed, in a recent antibiotic discovery project, *N. mucosa* was shown to display activity against other *Neisseria* in delayed antagonism assays, possibly through production of an antimicrobial secondary metabolite (Aho et al., 2020), revealing potentially novel and exploitable antagonism strategies among *Neisseria* species.

### Antagonism Mediated by Methylated DNA

Perhaps one of the most fascinating mechanisms of antagonism described among *Neisseria* species is the killing of *N. gonorrhoeae* by *Neisseria elongata* DNA. These species can interact intimately *via* Type four pili (Tfp) which permits bacteria: bacteria interactions and DNA exchange, including the transfer of resistance elements (Higashi et al., 2011). However, *N. elongata* can also inhibit gonococcal growth *in vitro via* a DNA-uptake dependent mechanism, as well as enhance clearance of gonococci in a mouse model of cervico-vaginal infection (Kim et al., 2019). The toxicity of DNA, released by *N. elongata* and taken up by the naturally competent gonococcus, was shown to be due to their different DNA methylation status which would result from their different repertoires of restriction modification systems (Kim et al., 2019). The proposed model by which subsequent cell death occurs postulates that due to the relatedness of the two species, the high degree of genomic DNA sequence homology allows for multiple foci of recombination to form following uptake; at these sites mismatched methylation leads to restriction enzyme cleavage and the eventual degradation of the chromosome (Kim et al., 2019; So and Rendón, 2019). These findings may have broader implications, as DNA from several commensals had the same effect, and *N. elongata* DNA was also shown to kill the meningococcus (Kim et al., 2019). Thus, this DNA based killing mechanism may be an important influence on the interactions among these related organisms.

Interestingly some gonococci and meningococci can also release DNA, *via* a Type IV secretion system (T4SS) (Dillard and Seifert, 2001; Hamilton et al., 2005) and by autolysis (Lappann et al., 2010), respectively. *N. meningitidis* extracellular DNA (eDNA) contributes to biofilm stability of certain lineages of *N. meningitidis* and this has been proposed to impact their colonization capacity. For example strains which are unable to use eDNA for biofilm formation (*i.e.*, those from the ST8 and ST11 lineages) may engage in more transient interactions with the host (Lappann et al., 2010). Therefore, extracellular *Neisseria* DNA could have multiple, contrasting influences on the ability of these organisms to successfully colonise the human nasopharynx. Also of interest, based on the model of Kim *et al*., is the fact that restriction modification systems undergo phase variation in *Neisseria gonorrhoeae* (Sánchez-Busó et al., 2019). Whether this variation could impact DNA-dependent antagonism among kin for example, is an interesting avenue to explore.

## Discussion

**Figure 2:**
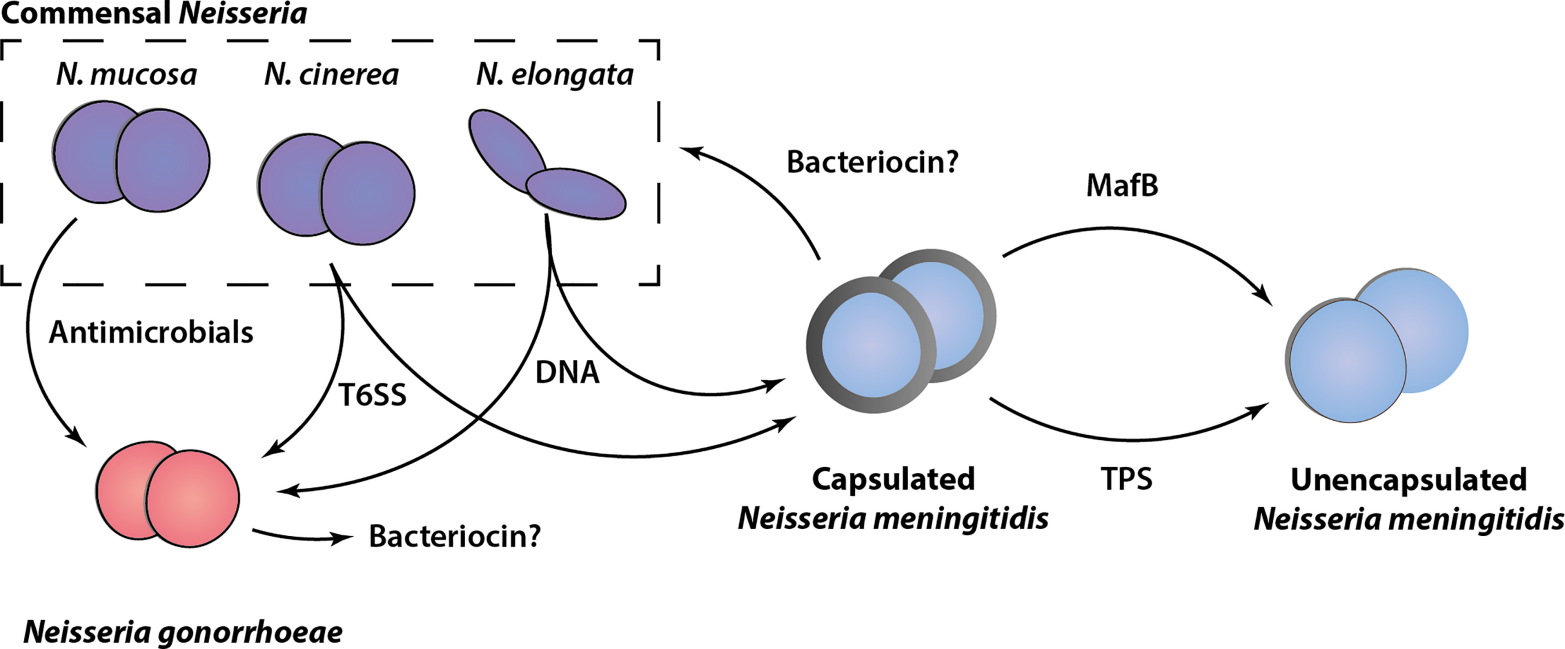
Schematic overview of antagonism systems in *Neisseria*. Experimentally validated growth inhibitory activities of different *Neisseria* species are shown. Commensal *Neisseria species* (*N. mucosa*, *N. cinerea* and *N. elongata*) are shown in purple, *Neisseria gonorrhoeae* (red), and *Neisseria meningitidis* (blue) in both capsulated (with grey outline) and unencapsulated form (without grey outline) are shown. The various mediators of growth inhibition/killing are indicated, with arrows pointing from the attacker to the prey cell based on current *in vitro* experimental evidence. Potential bacteriocins are indicated with a question mark.

As discussed in this review, several different growth inhibitory activities and mediators of bacterial antagonism have been identified among the members of the *Neisseria* genus. These include examples of well characterized contact-dependent bacterial antagonism mechanisms, such as the Tps systems and T6SS, less well-characterized activities for which the genetic basis or active components are as yet undefined (bacteriocins and antimicrobials) and a novel DNA-based killing mechanism. An overview of our current understanding of these growth inhibitory activities among *Neisseria* species is shown in **Figure 2**. Although this shows that both pathogenic and non-pathogenic *Neisseria* species are equipped with ways to inhibit potential competitors, in most cases these have been identified and analyzed in only a limited number of strains. Systematic genomic analysis and *in vitro* verification of these activities across and within species is lacking. In the case of polymorphic toxins and T6SSs for which the genetic components have been defined, interrogation of publicly available *Neisseria* species genomic sequence data [*e.g.*, PubMLST (Jolley et al., 2018)] using homology searches could provide insights into the distribution and diversity of the systems. In addition, online tools for identification of bacteriocin synthetic loci (Van Heel et al., 2018) may reveal candidate genes responsible for the growth inhibitory substances described in early studies.

Defining the distribution and conservation of the different systems would not only provide indication of the diversity and prevalence of these systems, but would also inform the judicious choice of strains for experiments to better understand the breadth of activity of antagonistic factors. For example, the effect of Mafs and TPS toxins have so far only been examined against isogenic strains that have been genetically engineered to lack immunity factors as well as other protective features such as capsule. Moreover, our understanding of these systems at both the molecular and functional level is limited. Key questions include how their expression and secretion is regulated in different strains, how toxins target and enter neighboring cells and the precise mechanism of toxicity. In particular, defining the role of the *Neisseria-*specific Maf toxins might shed light on novel modes of toxin secretion or function. Furthermore, given the sequence similarity in the C-terminal toxic domains of Maf proteins and Tps toxins (Arenas et al., 2015), the conserved modular architectures of the polymorphic toxins and the intriguing genetic locus arrangements (**Figure 1**), the possibility of interplay between these systems merits investigation.

Importantly, although this review has focused on antagonistic mechanisms that the *Neisseria* spp. use against each other, it is possible that these could be effective against other members of the microbiota, or host cells, as has been observed for some T6SS effectors (Monjarás Feria and Valvano, 2020). Defining toxin or antimicrobial targets may provide a clearer picture of their potential contribution to either *Neisseria* specific or more widespread bacterial competition. In addition, defining the nature and mode of action of toxins or secreted compounds could lead to development of novel antimicrobials, or help define novel targets, which is of particular interest for compounds with inhibitory or killing activity against the pathogenic *Neisseria* and in particular for *N. gonorrhoeae* which has developed high levels of antibiotic resistance (Unemo et al., 2019).

There remain challenges in extrapolating *in vitro* evidence of antagonism to its relevance for interactions between bacteria in the upper airway. Several of these antagonistic mechanisms are ‘contact-dependent’ systems which exert effects only on close neighbors. *Neisseria* species usually form aggregates known as microcolonies or biofilms mediated by Type four pili. *In vitro*, heterogeneity in Tfp can lead to spatial segregation within colonies, even of related strains (Oldewurtel et al., 2015; Zöllner et al., 2017; Pönisch et al., 2018) and this localized sorting can impact the efficacy of contact dependent systems (Custodio et al., 2021). The development of more complex *in vitro* (Łaniewski et al., 2017; Audry et al., 2019) and animal models (Yi et al., 2003; Weyand et al., 2013; Johswich et al., 2015; Ma et al., 2018) offers the promise of dissecting the specific contributions of potentially antagonistic factors in the establishment and stability of microbial communities. Clearly, the ground-breaking approach of challenging human volunteers with commensal species (Evans et al., 2011; Deasy et al., 2015) would provide the most physiologically relevant information, but is not suitable for high-throughput analysis and elucidation of molecular mechanisms.

Importantly, antagonistic interactions between these related bacteria might play a key role in shaping pathogen biology and behaviour. A key feature of the pathogens *N. meningitidis* and *N. gonorrhoeae* is their ability to adapt and undergo phase and antigenic variation. These features have resulted in them being highly adept at evading host immunity. Competitive interbacterial interactions may also be a driving force for variation with potential to impact factors implicated in virulence or colonization. Therefore, future work to understand the extent to which antagonistic relationships could drive variation or evolution of specific traits and alter pathogen physiology is warranted.

In summary, the upper respiratory tract can be regarded as a busy cross-roads for *Neisseria* spp., where pathogenic species may reside alongside a range of commensal *Neisseria* species. Both the meningococcus and some commensal species establish colonization lasting months, offering opportunity for co-operation and/or competition, which could impact the composition and shape of the community. In the future, it will be important to expand our emerging knowledge of antagonistic properties of these organisms and establish their underlying mechanisms. This will allow a better understanding of their roles in shaping interactions with each other and with the host.

## Author Contributions

RB, CT, and RE wrote and critically reviewed the manuscript. RB created figures with input from RE and CT. All authors approved the submitted version.

## Funding

Work in CT’s lab is supported by a Wellcome Trust Investigator award (102908/Z/13/Z) and the lab has received funding from Meningitis Now.

## Conflict of Interest

The authors declare that the research was conducted in the absence of any commercial or financial relationships that could be construed as a potential conflict of interest.

## Publisher’s Note

All claims expressed in this article are solely those of the authors and do not necessarily represent those of their affiliated organizations, or those of the publisher, the editors and the reviewers. Any product that may be evaluated in this article, or claim that may be made by its manufacturer, is not guaranteed or endorsed by the publisher.
